# Psychometric properties of outcome measures in non‐pharmacological interventions of persons with dementia in low‐and middle‐income countries: A systematic review

**DOI:** 10.1111/psyg.12647

**Published:** 2020-12-17

**Authors:** Bharath Du, Monisha Lakshminarayanan, Murali Krishna, Sridhar Vaitheswaran, Mina Chandra, Shaji Kunnukattil Sivaraman, Satyapal Puri Goswami, Thara Rangaswamy, Aimee Spector, Charlotte R. Stoner

**Affiliations:** ^1^ Foundation for Research and Advocacy in Mental health (FRAMe) Mysore India; ^2^ Schizophrenia Research Foundation Chennai India; ^3^ Foundation for Research and Advocacy in Mental health (FRAMe) Mysore India; ^4^ Schizophrenia Research Foundation Chennai India; ^5^ Atal Bihari Vajpayee Institute of Medical Sciences and Dr Ram Manohar Lohia Hospital New Delhi India; ^6^ Government Medical College Thrissur India; ^7^ All India Institute of Speech and Hearing Mysore India; ^8^ Schizophrenia Research Foundation Chennai India; ^9^ University College London London UK; ^10^ University of Greenwich London UK

**Keywords:** dementia, low middle income countries, non‐pharmacological therapy, outcome measures, psychometric properties

## Abstract

Despite high burden of dementia in low‐and middle‐income countries (LMICs), only a small number of clinical trials of psychosocial interventions for persons with dementia (PwD) have been conducted in these settings. It is essential that such trials use appropriate outcome measures that are methodologically robust and culturally appropriate to evaluate the effectiveness of interventions. We carried out a systematic review to examine the evidence base and psychometric properties of measures employed in these studies in LMICs. A systematic search of published literature on randomised controlled trials (RCT) of psychosocial interventions for PwD in LMICs between 2008 and April 2020 was carried out. Measures employed in each of the eligible studies were identified and through a focused search, we further explored the evidence base and psychometric properties employing Terwee criteria. Data extraction and quality appraisal were conducted by two independent reviewers. The review identified 41 measures from 17 RCTS which fulfilled eligibility criteria and they examined effectiveness across the domains of cognition (n = 16), behaviour and psychological symptoms (n = 11) and quality of life (n = 8). Of these 41, we were able to access relevant literature only for 18 and they were subject to psychometric analysis. Psychometric properties of these 18 instruments were at best modest, with Terwee scores ranging from 3 (low) to 15 (moderate). A majority of the studies were from China (n = 5) and Brazil (n = 6). The evidence base for the routinely employed measures in RCTs of non‐pharmacological interventions for PwD in LMICs is limited. The quality of adaptation and validation of these instruments is variable and studies are largely uninformative about their psychometric properties and cultural appropriateness to the study setting. There is an urgent need to develop scientifically robust instruments in LMIC settings that can be confidently employed to measure outcomes in trials of psychosocial interventions for PwD.

## INTRODUCTION

Demographic ageing is a global phenomenon and the most important social transformation of the 21st century.^1^ Of all the chronic non‐communicable diseases (NCDs) related to ageing, dementia and cognitive impairment are the leading contributors to disability, and particularly, dependence among older people worldwide.^2^

Worldwide, around 50 million people live with dementia, and this is estimated to reach 75 million by 2030.^3^ Two in three people with dementia live in low‐ and middle‐income countries (LMIC).^4^ This poses a huge challenge for governments to plan and design viable assessment and treatment options for persons with dementia suitable for their countries. In LMIC settings, dementia is often seen as part of normal ageing, is under‐recognised, under‐disclosed, under‐treated, and under‐managed.^5^ These factors make evaluation, treatment and research on dementia in these settings uniquely challenging, with specialist and culturally specific tools, methods for assessment and monitoring of treatment required.

Considering the aforementioned complexities and challenges of evaluation and treatment of dementia, the development of novel, tailor‐made therapeutic interventions is required for LMIC settings. Among all the interventions available, psychosocial interventions are particularly important and suitable as they are typically low cost and less resource intensive. They are more relevant to those settings where access to medicines and specialists is restricted. However, in this era of evidence‐based medicine, these novel psychosocial interventions need to be tested for their feasibility, efficacy and applicability in local contexts using gold standard randomised controlled trials (RCTs). Selecting appropriate outcome measures is a critical step in designing valid and useful clinical trials for persons with dementia, as the use of an unreliable measure may result in important information about the effectiveness of an intervention being lost or distorted.^6^

Choosing an appropriate outcome measure is even more important in LMIC settings as a significant number of measures used in intervention trials for persons with dementia were originally developed in high income countries (HICs). As there is little standardisation of methods for adaptation of these measures, their current ‘adaptation’ varies from cross‐cultural adaptation with adequate methodology to informal verbatim translation. There is no consensus as to which measures are most appropriate or psychometrically robust for use in persons with dementia.

The aims of this systematic review are to:

1. Identify outcome measures that are used to evaluate the effectiveness of psychosocial interventions for persons with dementia in LMICs
2. Conduct a quality appraisal of the psychometric properties of each of the outcome measures
3. Provide recommendations for use of outcome measures, based on their psychometric robustness.

## METHODS

### Design

**Figure 1:**
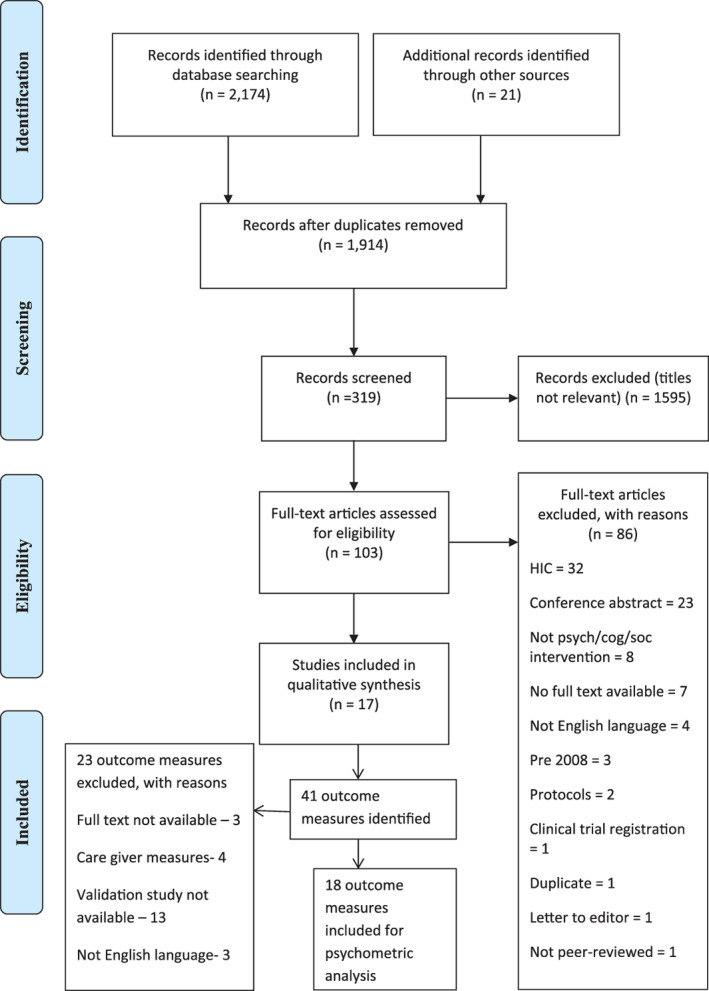
Process of systematic review (flow chart).

A systematic search of published literature from 2008 to 2019 on psychosocial interventions delivered to persons with dementia in LMICs was previously conducted by authors of this team.^7^ Results from this search consisted of 17 studies, describing 11 interventions in six countries. A repeat search was run in April 2020 using the published search strategy and the process of the systematic review is shown in Figure 1. Each of the studies included in this systematic review was subject to an additional search to identify relevant outcome measures used and focused searches were used to identify articles that described the development or adaptation of these measures for the countries in question. All included measures were subject to a quality appraisal to determine validity and reliability by employing Terwee criteria.^8^ This systematic review followed the standard Preferred Reporting Items for Systematic Reviews and Meta‐Analyses (PRISMA) guidelines for systematic review and a checklist for the same has been submitted as a Appendix S1 for further reading.^9^

### Search strategy

The full search strategy is described in a related review.^7^ Briefly, Cochrane Controlled Register of Trials (CENTRAL), PubMed, EMBASE, PsycINFO and MEDLINE were searched for studies published between 2008 and April 2020. Search terms fell under the categories: psychosocial or non‐pharmacological interventions, LMICs and people with dementia. LMICs were designated as such according to their classification by the Organisation for Economic Co‐operation and Development.^10^ The list of included studies was then examined and all outcome measures for cognitive, psychological and social domains were extracted. The reference list was then examined to identify articles that described the development of these measures for the country in question or, if it was an existing measure, the article that described the translation and adaptation of the measure for the country in question. These articles are subsequently described as ‘measure development or adaptation papers’ and were included regardless of publication date. In cases where the reference given was for an English language measure but not for the translated or adapted version, the corresponding author was contacted to ascertain which version of the measure was used.

### Inclusion and exclusion criteria

A measure development or adaptation paper was included if:

1. The domain measured was deemed by authors to be cognitive, psychological or social in nature
2. The outcome measure was used in an intervention study to examine change over a period of time in persons with dementia, as an indicator of benefit derived from the intervention
3. It was published in a peer‐reviewed journal.

A measure development or adaptation paper was excluded if:

1. It was published in a language other than English and an English translation was not available.

### Quality assessment

Included measures were grouped by domain (cognition, behaviour, depression, anxiety and quality of life) and a quality assessment was undertaken independently by two authors (BD and ML) against the Terwee criteria,^8^ used successfully in related reviews.^11^, ^12^ The Terwee criteria are based on a list of nine common psychometric properties: content validity, internal consistency, criterion validity, construct validity, reproducibility, responsiveness, floor and ceiling effects and interpretability and, for each domain listed, a maximum score of two was awarded if the psychometric properties were correctly evaluated and were within an acceptable range. A score of one per criterion was awarded if the methodology reported was flawed and zero was awarded if no information was reported or psychometric properties reported fell outside the acceptable range. Full criteria are provided in Table 1. After the initial appraisal, authors BD and ML discussed their ratings and any discrepancies until a consensus was reached.

**Table 1:** Terwee criteria

Property		Definition	Quality criteria	
1	Content validity	The extent to which the domain of interest is comprehensively sampled by the items in the questionnaire (the extent to which the measure represents all facets of the construct under question).	+2	A clear description of measurement aim, target population, concept(s) that are being measured, and the item selection AND target population (investigators OR experts) were involved in item selection.
			?1	A clear description of the above‐mentioned aspects in lacking OR only target population involved OR doubtful design or method.
			−0	No target population involvement.
			00	No information found on target population involvement.
2	Internal consistency	The extent to which items in a (sub)scale are inter‐correlated, thus measuring the same construct.	+2	Factor analyses performed on adequate sample size (7* # items and ≥ 100) AND Cronbach's alpha(s) calculated per dimension AND Cronbach's alpha(s) between 0.70 and 0.95
			?1	No factor analysis OR doubtful design or method
			−0	Cronbach's alpha(s) <0.70 or > 0.95, despite adequate design and method
			00	No information found on internal consistency
3	Criterion validity	The extent to which scores on a particular questionnaire relate to a gold standard	+2	Convincing arguments that gold standard is ‘gold’ AND correlation with gold standard ≥ 0.70
			?1	No convincing arguments that gold standard is ‘gold’ OR doubtful design or method
			−0	Correlation with gold standard <0.70, despite adequate design and method
			00	No information found on criterion validity
4	Construct validity	The extent to which scores on a particular questionnaire relate to other measures in a manner that is consistent with theoretically derived hypotheses concerning the concepts that are being measured	+2	Specific hypotheses were formulated AND at least 75% of the results are in accordance with these hypotheses
			?1	Doubtful design or method (e.g. no hypotheses)
			−0	Less than 75% of hypotheses were confirmed, despite adequate design and methods
			00	No information found on construct validity
5	Reproducibility			
5.1	Agreement	The extent to which the scores on repeated measures are close to each other (absolute measurement error)	+2	SDC < MIC OR MIC outside the LOA OR convincing arguments that agreement is acceptable
			?1	Doubtful design or method OR (MIC not defined AND no convincing arguments that agreement is acceptable)
			−0	MIC ≤ SDC OR MIC equals or inside LOA despite adequate design and method
			00	No information found on agreement
5.2	Reliability	The extent to which patients can be distinguished from each other, despite measurement errors (relative measurement error)	+2	ICC or weighted Kappa ≥ 0.70
			?1	Doubtful design or method
			−0	ICC or weighted Kappa <0.70, despite adequate design and method
			00	No information found on reliability
6	Responsiveness	The ability of a questionnaire to detect clinically important changes over time	+2	SDC or SDC < MIC OR MIC outside the LOA OR RR > 1.96 OR AUC ≥ 0.70
			?1	Doubtful design or method
			−0	SDC or SDC ≥ MIC OR MIC equals or inside LOA OR RR ≤ 1.96 or AUC <0.70, despite adequate design and methods
			00	No information found on responsiveness
7	Floor and ceiling effects	The number of respondents who achieved the lowest or highest possible score	+2	≤ 15% of the respondents achieved the highest or lowest possible scores
			?1	Doubtful design or method
			−0	>15% of the respondents achieved the highest or lowest possible scores, despite adequate design and methods
			00	No information found on interpretation
8	Interpretability	The degree to which one can assign qualitative meaning to quantitative scores	+2	Mean and SD scores presented of at least four relevant subgroups of patients and MIC defined
			?1	Doubtful design or method OR less than four subgroups OR no MIC defined
			00	No information found on interpretation

In order to calculate a total score + = 2;? = 1; − = 0; 0 = 0 (scale of 0–s18).

SDC, smallest detectable difference (this is the smallest within person change, above measurement error. A positive rating is given when the SDC or the limits of agreement are smaller than the MIC); MIC, minimal important change (this is the smallest difference in score in the domain of interest which patients perceive as beneficial and would agree to, in the absence of side effects and excessive costs); SEM, standard error of measurement; AUC, area under the curve; RR, responsiveness ratio; LOA, limits of agreement.

## RESULTS

Our search yielded 17 intervention studies from six LMICs. Studies were conducted in Brazil (n = 6), China (n = 5), India (n = 2), Tanzania (n = 2), Turkey (n = 1) and Argentina (n = 1). A wide range of interventions were evaluated: reality orientation, cognitive stimulation therapy (CST), reminiscence therapy, music therapy, tailored rehabilitation programs, games and other activities were used for the treatment of dementia. Forty‐one outcome measures were identified, of which 16 were primarily measures of cognition, three measured behavioural and psychological symptoms or distress in dementia, eight measured depression and anxiety, eight measured quality of life, four measured caregiver burden and two measured disability.

Only 18 outcome measures were included for psychometric analysis, as 23 had to be excluded for the following reasons: there was no evidence of validation or adaptation of the chosen outcome measure for the study setting (n = 13), inability to access the full articles (n = 3), scale measured other outcomes related to caregivers (n = 4) and the validation studies were not in English language (n = 3).

Most authors had provided the citation of the original development article of an outcome measure in English, but these studies lacked information related to cultural adaptation of the outcome measure for use in the study setting. For example, many authors referenced Folstein et al., 1975 for Mini Mental Status Examination (MMSE), which is an original development article.^13^ It is likely that many would have used a verbatim translated outcome measure (informal linguistic translation) instead of a systematically translated, adapted and validated measure (cultural adaptation) for study population and settings. Hence, we contacted the corresponding authors of all the eligible studies by email to obtain further clarification about the measures they had employed in their study, including the references for those measures. If no response was received after 2 weeks of initial contact, we sent them another email reminder. However, only three of the 17 authors (Li, Asiret and Camargo) replied. Li and colleagues had used linguistically translated (without formal adaptation and validation) outcome measures which were widely in use in China, while Asiret and Camargo had used culturally adapted and validated scales in Turkish and Portuguese languages respectively but had referenced original English developmental articles of the outcome measure. After discussing as a team, we decided to assume that authors who did not respond were likely to have used either a culturally adapted or verbatim translated version of the original outcome measure. Hence, for the purpose of this review, further searches were undertaken to identify the culturally adapted and validated version of measures specific to each country.

A summary of all the 17 intervention studies is given in Table 2. Psychometric properties of the outcome measures are described below and ratings of these measures based on Terwee criteria ARE tabulated in Table 3 and a further summary of their validation studies is given in Appendix S1.

**Table 2:** Summary of intervention studies

Sr. no	Study authors, country and language of study setting	Study description	Outcome measures used in the study	Evidence of adaptation or validation of outcome measure for study setting	Comments
1	Machado *et al*., 2009^14^ Brazil Portuguese	RCT to determine effect of participation of AD patients in a multidisciplinary rehabilitation program on cognition, depression and QOL	MMSE – Mini Mental State Examination	Yes (C)	No significant change in cognition from pre to post intervention in both experimental and control group.
			GDS‐Geriatric Depression Scale	Yes (C)	No significant change in depression scores from pre to post intervention in both experimental and control groups.
			QOL‐AD ‐ Quality of life in AD	Yes (A)	No significant change in QOL from pre to post intervention in both experimental and control groups.
2	Niu *et al*., 2010^15^ China Chinese	RCT to evaluate efficacy of cognitive stimulation therapy on individual dimensions of neuropsychiatric symptoms in AD patients	NPI ‐ Neuropsychiatric inventory	Yes (C)	Total score showed a significant improvement (−2.06 points, SE = 0.35) compared with a slight decline (0.00 points, SE = 0.26) in the control group (t¼ = −4.766, *P* < 0.001)
			MMSE – Mini Mental State Examination	Yes (C)	Significant improvement in score by 0.81 points, (SE = 0.28) in the treatment group and decline by 0.19 points (SE = 0.16) in the control group (t = 3.106, *P* = 0.004).
3	Viola *et al*., 2011^16^ Brazil Portuguese	To evaluate the effect of a multifunctional stimulation program on cognition, neuropsychiatric symptoms, and QOL in patients with mild AD in a controlled, single‐blind design	SKT ‐ Short Cognitive Test	Yes (A)	Significant increase in total scores of control group (*P* = 0.05) indicating deterioration of cognition in control group.
			QOL‐AD ‐ Quality of life in AD	Yes (A)	Significant improvement in scores (*P* = 0.004) in experimental group post intervention.
			NPI ‐ Neuropsychiatric inventory	Yes (C)	No significant change pre and post intervention in both groups.
			MMSE – Mini Mental State Examination	Yes (C)	No significant change pre and post intervention in both groups.
			GDS ‐ Geriatric Depression Scale	Yes (C)	Significant reduction in scores (*P* = 0.001) in experimental group post intervention.
4	Azcurra, 2012^17^ Argentina Spanish	RCT to evaluate the efficacy of reminiscence therapy in improving QOL in dementia patients	CDR – clinical dementia rating scale	No	
			CPS – cognitive performance scale	No	
			SES ‐ Social Engagement Scale	No	
			SRQoL ‐ the resident self‐reported	No	
			RAID ‐ rating anxiety in dementia	No	
			The Zarit Burden Interview	No	
			Minimum Data Set ‐ bad depression rating scale	No	
			Well‐being/ ill‐being scale	No	
			MMSE – Mini Mental State Examination	No	
			TSI – test for severe impairment	No	
5	Kumar *et al*., 2014^18^ India Hindi	RCT to evaluate the impact of a novel occupational therapy program on QOL of patients with mild to moderate dementia	WHOQOL – Bref WHO quality of life brief	Yes (A)	Significant improvement of scores (*P* < 0.001) post intervention in experimental group.
6	Lin *et al*., 2015^19^ China Chinese	To evaluate the impacts of a GO‐ game (Chinese chess) intervention on AD in a Northeast Chinese population and follow up evaluation at 6 months	MADRS‐ Montgomery‐Asberg depression rating scale	No	
			KICA Depression ‐ Kimberley Indigenous Cognitive Assessment of Depression	No	
			HADS‐ Hospital anxiety and depression scale	Yes (C)	A reduction of HADS mean score by 1.75 points (95% CI, 0.17–3.68) post intervention in experimental group.
			GAF ‐ Global assessment of functioning	No	
			RAND 36	Yes (C)	Statistically significant increase of 4.61 points (95% CI, −2.75–11.32) when compared with a control group (*P* < 0.05), providing evidence for efficacy of GO game program
			TAS 20 ‐ Toronto alexithymia scale	Yes (C)	No statistically significant difference between the experimental and control groups
7	Santos *et al*., 2015^20^ Brazil Portuguese	To evaluate the effects of a multidisciplinary rehabilitation program on cognitive ability, quality of life and depression symptoms in patients with AD and cognitive impairment without dementia (CIND).	MMSE – Mini Mental State Examination	Yes (C)	Significant increase in mean MMSE scores in both mild AD (*P* = 0.021) and CIND patients (*P* = 0.005).
			GDS ‐ Geriatric Depression Scale	Yes (A)	Mild AD patients (*P* < 0.001) and CIND patients (*P* = 0.011) in Experimental group had reduction in depressive symptoms
			QOL‐AD ‐ Quality of life in AD	Yes (A)	Mild AD patients who received the intervention had improvements in quality of life with significant increase in mean scores of QOL‐AD Brazil (*P* = 0.003)
8	Camargo *et al*., 2015^21^ Brazil Portuguese	To assess the effectiveness of reality orientation when combined with acetylcholinesterase inhibitors in the treatment of mild and moderate AD dementia.	CERAD neuropsychological battery	Yes (B)	Significantly higher mean CERAD score (*P* = 0.03) compared to control group after 6 months follow up
			MMSE – Mini Mental State Examination	Yes (B)	Treatment group scored significantly higher mean MMSE score (*P* = 0.03) compared to control group.
			CDT (clock drawing test)	No	Full text article not accessible
9	Asiret & Kapucu, 2015^22^ Turkey Turkish	To investigate the effect of reminiscence therapy on the cognitive status, depression, and daily living activities of institutionalised patients with mild and moderate AD	MMSE – Mini Mental State Examination	Yes (B)	Significant increase (*P* < 0.05) in mean r MMSE‐T score of therapy group compared to the control group
			GDS ‐ Geriatric Depression Scale	Yes (B)	Validation study in Turkish language, English translation could not be traced.
10	Raghuraman *et al*., 2017^23^ India Tamil	To culturally adapt, validate, and test the feasibility of delivering UK‐based CST as an acceptable intervention in Chennai, India	Feedback forms		Not a standard outcome measure
11	Paddick *et al*., 2017^24^ Tanzania Swahili	To conduct a trial of CST in a rural setting in Tanzania and evaluate its usefulness as a treatment for dementia using a stepped‐wedge design with randomisation	WHOQOL‐Bref ‐ WHO Quality of Life measure ‐ Brief	No	
			WHODAS 2 – WHO disability assessment scale	No	Disability assessment scale.
			ADAS‐COG – Alzheimer's Disease Assessment Scale – Cognitive subscale	Yes (A)	Significant improvement in cognition as evidenced by significant reduction in mean ADAS‐COG score after 8 weeks of therapy
			Barthel index ‐ activities of daily living	No	Dependency assessment scale
			HADS‐ Hospital anxiety and depression scale	No	
			Zarit Burden Interview	‐	Care giver burden scale
			NPI‐ Neuropsychiatric Inventory	‐	Carer rating was used in the study
12	Li & Li, 2017^25^ China Chinese	To investigate the efficacy of a Chinese folk recreational program on symptoms among older people with dementia in China	MMSE – Mini Mental State Examination	Yes (C)	Mean scores of MMSE increased significantly from baseline to week 16 (*P* < 0.01) in the experimental group, while for the control group, the mean score of MMSE decreased significantly (*P* < 0.01).
			NPI ‐ Neuropsychiatric Inventory	Yes (C)	Mean score of CNPI ‐ symptom decreased significantly (*P* < 0.01) in the experimental group
			Barthel index ‐ activities of daily living	No	
13	de Oliveira *et al*., 2018^26^ Brazil Portuguese	To investigate the effectiveness of the TAP intervention adapted for an outpatient memory clinic (tailored activity program–outpatient version (TAP‐O)) on reducing NPS and caregiver burden in patients with dementia.	NPI ‐ Neuropsychiatric Inventory	Yes (A)	Treatment group had a significant decrease in hallucination (*P* = 0.04), agitation (*P* = 0.03), anxiety (*P* = 0.02), aggression (*P* = 0.01), sleep disorder (*P* = 0.02), aberrant motor behaviour (*P* = 0.02)
			Zarit Burden Interview	No	Care giver burden measure
14	Lyu *et al*., 2018^27^ China Chinese	To explore the effects of music therapy on cognition, BPSD, and ADL of AD patients and their caregiver distress	MMSE – Mini Mental State Examination	Yes (C)	No significant difference in scores in experimental group pre and post intervention
			WHOUCLA AVLT‐ The WHO University of California‐Los Angeles, Auditory Verbal Learning Test	No	Validation study in Chinese language was found, but English translation could not be traced.
			The semantic verbal fluency test	No	
			NPI ‐ Neuropsychiatric Inventory	Yes (C)	Experimental group scored significantly lower compared to control group post intervention
			Zarit Burden Interview	No	
15	Mkenda *et al*., 2018^28^ Tanzania Nigeria	To describe the adaptation and feasibility assessment of CST as a potential low‐resource intervention for dementia in Tanzania and Nigeria.	WHOQOL ‐ WHO Quality of Life measure	No	
			ADAS‐COG – Alzheimer's Disease Assessment Scale – Cognitive subscale	Yes (C)	Data were not analysed to assess improvements in outcome due to the small number of participants involved in the study
16	Novelli *et al*., 2018^29^ Brazil Portuguese	To evaluate the effect of TAP‐BR (tailored activity program ‐ Brazil) on the number, frequency, and intensity of BPSD and QOL of persons with dementia	NPI ‐ Neuropsychiatric Inventory	Yes (A)	Experimental group had significantly reduced total NPI score (*P* = 0.00; Cohen d = 0.95), number (*P* = 0.00; Cohen d = 0.93), frequency (*P* = 0.00; Cohen d = 1.12), intensity (*P* = 0.00; Cohen d = 0.77) of Behavioural and Psychological Symptoms in Dementia (BPSD)
			Zarit Burden Interview		Care giver burden measure
			QOL‐AD ‐ Quality of life in AD	Yes (A)	Caregivers in the experimental group had reported improvement in their own QOL (*P* < 0.05; Cohen d = 0.57) and that of the person with dementia (*P* < 0.01; Cohen d = 0.56). No differences were found in the ratings of QOL by the person with dementia themselves.
17	Wang *et al*., 2018^30^ China Chinese	To evaluate effects of music therapy on cognitive function and behaviour of mild AD patients receiving pharmacological intervention	MMSE – Mini Mental State Examination	Yes (C)	Patients in the music therapy group demonstrated a significant improvement (*P* = 0.003) over patients in the control group
			NPI ‐ Neuropsychiatric Inventory	Yes (C)	Patients in The music therapy group demonstrated a significant improvement in NPI scores (*P* < 0.01) over patients in the control group
			MoCA ‐ Montreal Cognitive Assessment	Yes (C)	Patients in the music therapy group demonstrated a significant improvement (*P* < 0.01) over patients in the control group

A, provided by the authors in the study; B, provided by authors after request for clarification by Email; C, found by focused search.

**Table 3:** Psychometric rating of outcome measures based on Terwee criteria

Construct	Outcome measure version	Content validity	Internal consistency	Criterion validity	Construct validity	Agreement	Reliability	Responsiveness	Floor and ceiling effects	Interpretability	Total
Cognition	MoCA‐BC Chinese^31^	+2	?1	+2	+2	?1	+2	+2	+2	?1	15
Cognition	ADAS‐Cog SSA^32^	+2	?1	+2	00	00	+2	+2	00	?1	10
Cognition	r MMSE‐Turkey^33^	+2	?1	?1	00	+2	+2	+2	00	00	10
Cognition	SKT‐Brazil^34^	+2	?1	+2	?1	00	00	+2	00	?1	9
Cognition	CERAD‐Brazil^35^	+2	00	00	+2	00	00	+2	00	?1	7
Cognition	TAS 20‐Chinese^36^	+2	+2	00	?1	+2	00	00	00	00	7
Cognition	MMSE‐Brazil^37^	+2	00	?1	00	00	00	+2	00	?1	6
Cognition	CAMSE^38^	+2	00	?1	00	+2	00	00	00	?1	6
BPSD	NPI‐Brazil^39^	+2	?1	00	00	+2	+2	00	?1	?1	9
BPSD	NPI‐clinician ‐ Brazil^40^	+2	00	?1	+2	00	+2	00	00	?1	8
BPSD	NPI Chinese^41^	+2	+2	00	?1	+2	00	00	00	00	7
Depression and anxiety	HADS Chinese^42^	+2	?1	?1	?1	00	00	+2	00	?1	8
Depression	GDS‐30 Brazil^43^	+2	?1	+2	00	00	00	+2	00	00	7
Depression	GDS‐15 Brazil^44^	+2	00	+2	00	00	00	+2	00	00	6
QOL	RAND Chinese^45^	+2	+2	?1	+2	+2	00	00	00	?1	10
QOL	WHOQOL‐Bref^46^	+2	?1	?1	+2	+2	00	00	00	00	9
QOL	QOL‐AD‐Brazil^47^	+2	?1	+2	+2	00	00	00	00	?1	8
QOL	WHOQOL‐Hindi^48^	+2	?1	00	00	00	00	00	00	00	3

BPSD, Behavioural and Psychological Symptoms of Dementia; QOL, quality of life.

## OUTCOME MEASURES RELATED TO COGNITION

A total of eight scales that measure cognition were included. The Chinese Montreal Cognitive Assessment Basic (MoCA‐ BC) scored most robustly on psychometric properties with a score of 15/18. Alzheimer's Disease Assessment Scale – Cognitive sub‐Saharan Africa (ADAS‐Cog SSA ‐ 10/18), the Revised Turkish MMSE (r MMSE –Turkish‐ 10/18) and the Short Cognitive Test (SKT Brazil version – 9/18) showed moderate score on psychometric analysis while Toronto Alexithymia Scale 20 Chinese (TAS 20 Chinese ‐ 7/18), Consortium to Establish a Registry for Alzheimer's Disease‐ neuropsychological battery (Consortium to Establish a Registry for Alzheimer's Disease (CERAD) Brazil ‐ 7/18), MMSE‐ Brazil (6/18) and Chinese adapted MMSE (6/18) scored poorly.

### Chinese version of MoCA‐BC

The MoCA basic (MoCA‐B) was developed by Nasreddine (1996) in Canada to screen for MCI in illiterate individuals and those with little education.^31^, ^49^ The Chinese version of the MoCA‐B (MoCA‐BC) was translated from the original English version. The MoCA‐BC was reported to have good content validity and criterion‐related validity (Pearson correlation coefficient of MoCA‐BC vs. MMSE = 0.787) and reliable internal consistency (Cronbach's α = 0.807). This scale showed good responsiveness, with the area under the receiver operating characteristic (ROC) curve more than 0.8 across all education levels in Chinese older adults. Inter‐rater reliability was also excellent with intraclass coefficient value of 0.96 (*P* < 0.001).^31^

### Alzheimer's Disease Assessment Scale – Cognitive subscale for sub‐Saharan Africa‐ ADAS‐Cog SSA

The ADAS‐Cog was developed in the 1980s to assess the level of cognitive dysfunction in Alzheimer's disease (AD), but its use has extended into pre‐dementia studies despite concerns about its ability to detect important changes at these milder stages of disease progression.^32^, ^50^ One team adapted the ADAS‐Cog for use in sub‐Saharan African settings with low literacy levels. The area under the ROC curve as 0.973 (95% CI = 0.936–1.00) for dementia, indicating good responsiveness of the scale. Internal consistency was high (Cronbach's α = 0.884) and inter‐rater reliability was excellent (intraclass correlation coefficient (ICC) 0.905, 95% CI 0.804–0.964). The scale also showed excellent content and criterion validity with convincing arguments and demonstration of strong correlation with severity of dementia measured with Clinical Dementia Rating Scale (CDR).^32^

### Short Cognitive Performance Test Brazilian version‐SKT Brazil

The SKT is a bedside cognitive screening battery designed to detect memory and attention deficits.^34^, ^51^ Flaks and colleagues have validated a Brazilian version of the SKT and reported the area under ROC ranging between 0.7 and 1, suggesting that the SKT adequately discriminates AD from participants without dementia (MCI and controls), irrespective of education. Inter‐rater and test–retest agreement, floor and ceiling effects were not reported by the authors. However, authors have mentioned that the preliminary study in Brazil showed good internal consistency, with Cronbach's α equal to 0.8 and significant correlation with MMSE and the CDT (clock drawing test).^34^

### Toronto Alexithymia Scale 20 Chinese version ‐TAS 20‐Chinese

Bagby and colleagues developed TAS 20 in 1994 from an earlier 26 item version developed by them.^36^, ^52^ It has three subscales: Difficulty Describing Feelings to others (DDF), Difficulty Identifying Feeling (DIF) and Externally‐Oriented Thinking (EOT) designed to measure deficiency in understanding, processing, or describing emotions. Zhu and colleagues translated the TAS to Chinese with involvement of Chinese psychologists and developers of the original English TAS and reported good content validity. Confirmatory factor analysis was conducted, which showed that a three factor model showed best acceptable standards and a Cronbach's alpha >0.7 showed high internal consistency. Test–retest coefficient for the whole scale together and subscales were >0.7 showing good test–retest reliability. However, there was no information on criterion validity, construct validity, responsiveness, floor and ceiling effects or inter‐rater agreement in the article.^36^

### CERAD – Brazil version

The CERAD was funded in 1986 by the National Institute on Ageing to develop a standardised assessment tool of AD for use by all Alzheimer Disease Centres established in the United States.^35^, ^53^ It consists of a clinical battery, neuropsychological battery, neuroimaging battery, family history scale, behavioural problems scale, family history assessment, services assessment, autopsy resources and educational brochures. Bertolucci and colleagues evaluated its validity in Brazil and reported that all the tests in CERAD had good sensitivity and specificity ranging from 73–97% and 67–87% respectively, with the exception of the Boston Naming Test with sensitivity of 61% and Constructional Praxis with specificity of 51%. All the tests showed good responsiveness with areas under ROC curve ranging between 0.7 and 0.9. However, internal consistency, criterion validity, test–retest reliability, inter‐rater reliability, floor and ceiling effects have not been reported.^35^

### MMSE

Folstein and his colleagues formulated the MMSE, a 30‐point psychological tool for measuring cognitive impairment.^13^ Since then it has been adapted to multiple languages and regions and extensively used in clinical and research settings.^54^ In this review, three culturally adapted MMSE scales were evaluated.

### Revised MMSE Turkey version – r MMSE‐T

The authors reported areas under ROC curve in educated and uneducated older people to be 0.953 and 0.907 respectively, which indicates good responsiveness of the outcome measure in detecting clinically important change in cognitive function over time.^33^ The scale had good content validity, internal consistency, inter‐rater and intra‐rater agreement with Cronbach's α and kappa values higher than 0.7 for both educated and uneducated older people. Cut‐off point of 22/23 of r MMSE‐T in the educated older people had the highest sensitivity (90.9), specificity (97.0) and positive likelihood ratio (30.3), whereas cut‐off point of 18/19 of the test in uneducated older people had the highest sensitivity (82.7%), specificity (92.3%) and positive likelihood ratio (10.7). Construct validity, floor and ceiling effects of the scale have not been reported.^33^

### MMSE‐Brazil version ‐ MMSE‐Brazil

A modified translated Portuguese version of the MMSE, proposed by Bertolucci and colleagues in 1994 and Almeida and colleagues in 1998 was used in this validation study.^37^, ^55^, ^56^ The authors involved geriatricians in item selection during measure adaptation and reported good content validity. Sensitivity, specificity, positive and negative predictive values were 80.8%, 65.3%, 44.7% and 90.7% respectively for a cut‐off point of 23/24. The area under the ROC curve was 0.807, indicating good responsiveness. Criterion validity has been tested with diagnosis of dementia by geriatricians using structured interviews based on Diagnostic and Statistical Manual 4^th^ edition (DSM‐IV) and International Classification of Diseases Edition 10 (ICD‐10). However, information on other psychometric measures such as internal consistency, inter‐rater agreement, test–retest reliability, construct validity, responsiveness and floor and ceiling effects was lacking.^37^

### Chinese adapted MMSE – CAMSE

The CAMSE was adapted from the original MMSE with some changes in test items to minimise literacy dependency and render them compatible with Chinese culture, while the main structures of the original test were kept intact and similar principles for scoring were used as much as possible.^38^ This suggests that the CAMSE tests the same cognitive functions as the original MMSE. Literate participants scored a higher CAMSE total score than illiterate participants (*P* < 0.05) to yield optimal cut‐off points of 22 for literates and 20 for illiterates with a sensitivity of 83.87% and a specificity of 84.48%. Corresponding positive predictive value (PPV) was 0.65, and negative predictive value (NPV) was 0.94. The test–retest reliability tested after 4–6 weeks for total scores was 0.75 (*P* < 0.01). However, the article lacked information on internal consistency, criterion validity, construct validity, responsiveness, floor and ceiling effects and interpretability of the scale.

## OUTCOME MEASURES RELATED TO BEHAVIOURAL AND PSYCHOLOGICAL SYMPTOMS IN DEMENTIA (BPSD)

### NPI ‐ Neuro‐Psychiatric Inventory

The NPI is a tool which measures behavioural disturbances in dementia using two separate scales for rating the severity of each symptom and the distress caused to the caregiver respectively.^57^ It was originally developed by Cummings *et al*. in 1994.^57^ This scale was used in eight studies across three countries – Brazil, China and Tanzania. Adaptation studies of NPI Brazil and China versions are reviewed here, while adaptation to Tanzania could not be traced.

### NPI ‐ Brazil

This tool received a Terwee score of 9 and reported test–retest reliability (Spearman's rho for total severity = 0.82), internal consistency (Cronbach's α = 0.7 for both severity and distress scales) and inter‐rater reliability (ICC severity = 0.98, distress = 0.96).^39^ It also provided ample information on content validity for the Portuguese translation, and both ceiling and floor effects for some items. It was one of the few papers that provided some information on floor and ceiling effects. However, it was uninformative on criterion validity, construct validity or responsiveness.^39^

### NPI ‐ Brazil‐ clinician version

This adapted tool scored 8/18 on the Terwee scale as it lacked information on internal consistency, agreement, responsiveness and floor and ceiling effects.^40^ The validation focused mostly on inter‐rater reliability (ICC of 0.923) and convergent validity with seven other scales, each of which measure various behavioural problems in dementia, with a sample of 156 participants. Convergent validity with the Apathy Inventory, Cohen‐Mansfield agitation index, Cornell Scale for depression in dementia and Brief Psychiatric Rating scale – delusions was high (Pearson correlation r ≥ 0.7) but was poor with Brief Psychiatric Rating Scale – hallucinations (r = 0.432). Even though the authors mentioned conducting test–retest reliability analysis, the results were not reported in the paper.^40^

### Chinese NPI – CNPI

This tool scored 7 on the Terwee scale and had clear information on content validity, internal consistency (Cronbach's α = 0.69 for the severity and 0.72 for the caregiver distress scale) and agreement (test–retest correlation coefficient between 0.66 and 0.98).^41^ Construct validity was also analysed through the Kaiser‐Meyer‐Olkin value which confirmed that there were five common factors present within the tool. Of note, there were no clear hypotheses tested in the paper.^41^

## OUTCOME MEASURES RELATED TO DEPRESSION AND ANXIETY

### Hospital Anxiety and Depression Scale ‐ Chinese version ‐ HADS Chinese

The HADS was originally developed by Zigmond and Snaith (1983) to screen for depression and anxiety in general hospital patients.^42^ Leung and colleagues validated a Chinese‐Cantonese version of the HADS against the Hamilton Rating Scale of Depression (HRSD) and Hamilton Rating Scale of Anxiety (HRSA) and reported good internal consistency (Cronbach's α = 0.86) and concurrent validity (Pearson's coefficient = 0.67 and 0.63, respectively; *P* < 0.001) with favourable sensitivity (0.79; 95% CI = 0.66–0.90) and specificity (0.80; 95% CI = 0.69–0.91) for screening for psychiatric disorders. However, its performance was marginally inferior to that of the HRSD. The authors did not report test–retest reliability, inter‐rater agreement, floor and ceiling effect and hence scored moderately (8/18) on Terwee criteria. As the validation has been done in a general population, this questions its applicability in dementia research.^42^

### Geriatric Depression Scale Brazil ‐ 30 item version ‐ GDS 30 Brazil and 15 item version ‐ GDS 15 Brazil

GDS was originally developed in 1983 by Yesavage.^43^, ^44^, ^58^ The original 30 item version of the GDS has been shortened and separately adapted and validated into scales with 15, 10, four and one item(s) across many languages and cultures.

Two studies from Brazil have validated the GDS for use in the local community. Paradela and colleagues validated the shortened GDS‐15 version with a geriatric population.^44^ This study obtained a Terwee score of 6/18, while Castelo et al. validated GDS 30 and scored 7/18 points on the Terwee scale. Both studies reported on the content validity adequately (with description on translation and back translation by experts), criterion validity (against DSM‐IV criteria based diagnosis provided by a trained clinician) and responsiveness (area under the curve value above 0.9).^43^, ^44^ Castelo *et al*. validated all versions of the GDS (30, 15, 10, four and one) and additionally reported on internal consistency (Cronbach's α = 0.7 or above in all of the tools).^43^ Both lacked information on construct validity, test–retest and inter‐rater agreement, floor and ceiling effects and interpretability.^43^, ^44^

## OUTCOME MEASURES RELATED TO QUALITY OF LIFE (QOL)

Four measures examined QOL in persons with dementia. The Chinese Short Form health survey‐36 (SF‐36) scored 10/18 and World Health Organization QOL assessment scale brief (WHOQOL‐ BREF) scored 9/18, while the Brazilian version of the QOL ‐ Alzheimer's disease (QOL‐AD) scored 8/18 and WHOQOL‐Hindi scored only 3/18.

### Chinese Short Form health survey‐36 ‐ SF‐36/ RAND 36 Chinese

The SF‐36 was developed as part of a medical outcomes study.^45^, ^59^ Li *et al*. in 2003 adapted and validated it for Chinese use. The content validity was found to be good with a clear description of measurement aim, target population, concept being measured and involvement of target population in item selection. Convergent validity and discriminant validity were satisfactory for all except the social functioning scale. The Cronbach's α coefficient ranged from 0.72 to 0.88 except 0.39 for the social functioning scale and 0.66 for the vitality scale. Test–retest reliability coefficients (at 2 weeks) ranged from 0.66 to 0.94. Factor analysis identified two principal components explaining 56.3% of the total variance. Inter‐rater reliability, responsiveness and floor and ceiling effects were not reported.^45^

### QOL for patients with AD Brazilian version ‐ QOL‐AD Brazil

Logsdon *et al*. proposed the QOL‐AD, which has three versions: two addressing the patient's QOL: one for the patient himself/herself (PQOL) and another for the caregiver perception of patient's QOL‐ CPQOL), and a third related to the QOL of the Caregiver‐ (CQOL).^47^, ^60^ The QOL‐AD has been translated and adapted to Portuguese by Novelli *et al*. Authors reported Cronbach's α of more than 0.8 for all the three versions. Content validity and construct validity were found to be good with convincing arguments for the same. Criterion validity was not determined as there was no instrument available for evaluation of QOL in dementia in Portuguese. The authors did not report test–retest and inter‐rater reliability, responsiveness, floor and ceiling effects.^47^

### WHO QOL assessment scale brief ‐ WHOQOL‐BREF and WHOQOL‐Hindi

WHOQOL‐BREF has been derived from the WHOQOL‐100 tool, which was developed by the WHOQOL Group in 15 international field centres as a cross‐culturally applicable QOL assessment tool.^46^, ^48^, ^61^, ^62^ The authors reported high correlations ranging from 0.89 to 0.95 between domain scores based on the WHOQOL‐100 and WHOQOL‐BREF. Cronbach's α ranged from 0.66 to 0.84 demonstrating good internal consistency. Content validity and test–retest reliability (range from 0.66 to 0.87) was good, while discriminant validity was excellent. However, inter‐rater reliability, responsiveness and floor and ceiling effects were not reported.^46^

WHOQOL‐Bref Hindi was developed in Delhi, one of the 15 centres in the WHOQOL study. The authors reported that the Hindi version and other national versions were compatible and comparable, as the WHOQOL was developed simultaneously in many centres across the world. However, the article was uninformative about the psychometric properties of WHOQOL‐Bref Hindi.^48^

## DISCUSSION

Eighteen outcome measures related to persons with dementia were identified (covering the constructs of cognition, behavioural and psychological symptoms, QOL, anxiety and depression) from 17 psychosocial intervention studies in LMICs. All of these were culturally adapted and validated versions from an original English measure, indicating a lack of indigenously developed measures in the native language/s of LMIC. Most measures achieved a modest score on their adaptation procedures, with the MoCA‐Chinese version scoring highest (15/18) and the WHOQOL‐Bref Hindi scoring the lowest (3/18) on Terwee criteria.

In intervention studies involving persons with dementia, the most commonly employed indicators of effectiveness are measures of cognition. Of the nine outcome measures for cognition, the MoCA‐BC (Chinese) was the most robustly developed, while the SKT Brazil version, ADASCOG‐SSA and r MMSE‐T gave moderate results on psychometric analysis. These tools appear to be adequate measures of cognition in patients with dementia. The TAS 20 Chinese version, CERAD Portuguese version, CAMSE and MMSE‐Brazil version scored low on psychometric analysis and need further psychometric examination before they can be used routinely. All the cognition measures were validated in geriatric populations except TAS 20 Chinese version, which has been validated in undergraduate students.

BPSD form another important dimension of dementia research. The NPI is one of the most widely used tools for evaluating BPSD and all the three versions ‐ NPI Brazil clinician version, NPI Brazil version and NPI Chinese version ‐ have been developed with moderate robustness and are adequate to detect and measure BPSD. However, further adaptation and validation of NPI to other languages and regions of LMIC is essential. The HADS Chinese, GDS 30 Brazil and GDS 15 Brazil used to measure anxiety, depression in hospital patients and depression in geriatric population respectively, have been developed with moderate robustness. However, the HADS Chinese is validated for general hospital patients and its validity for research in dementia is questionable and requires further psychometric examination before it can be routinely used with confidence.

Quality of life is a more recent but firmly established theme in dementia research, facilitating an integrative model for dementia treatment and care. The QOL‐AD Brazil, WHOQOL‐Bref and Chinese SF‐36 appear to be adequate measures of QOL, while WHOQOL‐Bref Hindi appears to be a poor measure of QOL as the authors did not report most psychometric parameters. The Chinese SF‐36 and WHOQOL have been validated in general populations and their validity for research in dementia is questionable and requires further psychometric examination before they are routinely used.

Many studies had not used adequate methodology for transcultural adaptation of an outcome measure, instead used an informally translated measure for validation. Cultural adaptation of a tool involves the production of an equivalent instrument for a target population, one that measures the same phenomenon in the original and the target cultures, rather than a verbatim translation. The first phase of the process includes a translation of words and sentences from the original language to another and then further linguistic adaptation to the cultural context of the target population to ensure that the new version is conceptually and culturally pertinent. The second phase of the cultural adaptation includes a validation phase during which the instrument is proven to be psychometrically equivalent to the original version.^63^, ^64^ Even when translated versions are in a population's native language, there can be cultural differences in the verbal expression of concepts, in meaning, and in relevance that may affect confidence in the validity of results obtained using the translation.^65^ Furthermore, a verbatim translated measure of cognition would increase the possibility of false positive rates of dementia as participants undergoing the test might skip or give wrong answers due to lack of understanding of the questions and alien concepts of the test, rather than cognitive deficits. This highlights the need for use of transcultural adaptation of outcome measures with adequate methodology in place of informal linguistic translations.

## METHODOLOGICAL ISSUES AND LIMITATIONS

All the measures included here failed to define minimal important change, which is a requisite of Terwee criteria for interpretability and responsiveness. Except for MoCA‐BC, no other validation study reported the floor and ceiling effects. This meant scoring the measures for interpretability and floor and ceiling effect was nearly impossible. Even though most authors reported Cronbach's alpha, they failed to report information on factor analysis performed on adequate sample size, leading to poor scores on internal consistency. We also noted that many authors had reported sensitivity, specificity, PPV and NPV in their validation papers, but these statistical tools are not included in the Terwee criteria. This suggests that researchers consider sensitivity and specificity as important tools to be tested in a validation study and further hints toward the need for a more inclusive and comprehensive psychometric criterion, which includes sensitivity and specificity of outcome measures in the psychometric analysis.

Referencing in scientific literature is very important as it gives the readers an understanding of the source of the information and also enables them to find the source of information for further reading if necessary. However, if the standard guidelines for referencing are not adhered in scientific articles, it undermines the purpose of referencing. In this review, we found many researchers citing the reference of an original development article of the outcome measure instead of the actual culturally adapted and validated version used in the research work in the country in question. Furthermore, some validation studies for these outcome measures were difficult to locate and could only be located by extensive searching. Also, many outcome measures had to be excluded from the review as adapted versions could not be found despite exhaustive searching. This warrants a need to promote and sensitise researchers about standard referencing guidelines.

Although we employed broad search criteria to identify potentially eligible studies, it is still possible that we may have missed out some studies due to heterogeneous nature of reporting changes in psychosocial interventions studies among persons with dementia.

## IMPLICATIONS FOR RESEARCH AND PRACTICE

Our review highlights the need for researchers to examine and ensure appropriate psychometric properties of outcome measures to be included in their research, while designing the research protocol and use outcome measures designed for a specific population, for a particular age group, region, culture and language to avoid skewed results and for better applicability of results in the population in question. Researchers should also provide references to the specific adapted version of an outcome measure correctly, in addition to referencing an original outcome measure developed in a different study setting. This review highlights limited availability of indigenously developed, culturally appropriate and validated outcome measures in LMIC, which may have inadvertently led the investigators of the studies included in this systematic review to use verbatim translated instruments. Even though most studies included in this review reported statistically significant effect of the intervention across domains of cognition, psychological symptoms and QOL, little is known about its clinical effectiveness.

This review indicates that MoCA‐BC (for cognition) and Chinese SF‐36 (for QOL), SKT Brazil version (for cognition) and NPI Brazil (for BPSD), ADASCOG‐SSA (for cognition) and r MMSE‐T (for cognition) can be used in dementia research with confidence in China, Brazil, sub‐Saharan Africa and Turkey respectively. Researchers should be aware of lack of psychological robustness of other outcome measures evaluated here. We suggest researchers exercise caution about the psychometric properties of outcome measures while choosing outcome measures for their research pursuits and, also while interpreting results of an intervention study from a LMIC setting. LMICs are characterised by populations with distinctively different cultures and spoken languages that are specific to a region within a country, which limits the generalisability and applicability of outcome measures and results of an intervention study beyond the study setting. Therefore, the first step in planning an intervention study for persons with dementia in LMICs should be to develop culture and context specific measures in their language/s and establish their psychometric properties.

## CONCLUSION

The evidence base for the routinely employed measures in RCTs of non‐pharmacological interventions for persons with dementia in LMICs is limited. The quality of adaptation and validation of these instruments is variable and studies are largely uninformative about their psychometric properties and cultural appropriateness to the study setting. There is an urgent need to develop scientifically robust instruments in LMIC settings that can be confidently employed to measure outcomes in trials of psychosocial interventions for persons with dementia.

## Supporting information


**Appendix S1:** Supplementary Information.
