# Supplementary material for: Psychometric properties of outcome measures in non‐pharmacological interventions of persons with dementia in low‐and middle‐income countries: A systematic review
Source: Psychogeriatrics. 2020 Dec 17;21(2):220–38. doi: 10.1111/psyg.12647 (PMC7986620; doi:10.1111/psyg.12647)
Supplement: Supplementary file 1 — Appendix S1: Supplementary Information. [file PSYG-21-220-s001.zip › PSYG_12647_supplementary file 2-outcome measure validation studies summary.docx]

| **Construct and Instrument** | **Interventional**  **Studies in which the outcome measure has been used** | **Validation study authors**  **and**  **Country and language** | **Description of outcome measure** | **Sample population** | **Methods** | **Reliability** | **Validity** |
| --- | --- | --- | --- | --- | --- | --- | --- |
| Cognition  MoCA-BC  Chinese version | Wang et al., 2018 | Chen et al., 2016  China  Chinese | -30 point scale with nine cognitive domains (executive function, language, orientation,  calculation, conceptual thinking, memory, visuoperception,  attention, and concentration)  -higher the scores, better is the cognition | 704 individuals, 280 cognitively  normal controls, 264 individuals with MCI, and 160 individuals with mild AD. | Cross sectional study To evaluate the effectiveness of MoCA-BC as a screening tool for detecting mild cognitive impairment (MCI) in Chinese elderly adults and validate it against MMSE. | - Cronbach alpha = 0.807  - ICC for inter-rater reliability was 0.96 (P < .001). | -Pearson correlation coefficient MoCA-BC v/s MMSE = 0.787  - cutoff 19 for low education group (≤6 years) with sensitivity= 87.9%, specificity= 81.0%, AUC= 0.896; 22 for the middle education group (7–12 years)  (sensitivity=92.9%, specificity=91.2%, AUC=0.949); and 24  in the high education group (>12 years) (sensitivity=89.9%, specificity=81.5%, AUC=0.916) |
| Cognition  ADAS cog- SSA | Paddick et al., 2017  Mkenda et al., 2018 | Paddick et al., 2017  Tanzania  Nigeria | 11 items scale, scored out of 70, with lower scores indicating less impairment.  Three main subscales are; language,  memory and new learning, and praxis | 34 individuals with dementia and 32 controls selected from community screening. | Cross section study to adapt the ADAS-Cog for use in  low-literacy settings in Sub Saharan Africa and conducted a feasibility  study of this low-literacy ADAS-Cog in individuals  with and without dementia in rural Tanzania | -Cronbach’s α was between 0.737 - 0.711 for the 2 groups and 0.884 for the whole cohort.  - ICC was 0.905 (95% CI 0.804–0.964) for total ADAS-Cog  Score, 0.977 (95% CI 0.945–0.991) for language subscores, 0.956 (95% CI 0.895–0.982) for praxis sub scores, 0.730 (95% CI 0.520–0.901) for memory subscale | - Concurrent  Validity assessment demonstrated very high correlation  between the ADAS-Cog score and the IDEA cognitive screen score (r = 0.833, p<0.001) and CDR score (r = 0.795, p<0.001) |
| Cognition  Revised Mini Mental State Examination Turkish  (r MMSE T) | Asiret et al., 2015 | Keskinoglu  et al., 2009  Turkey    Turkish | 30 point questionnaire with questions to check orientation to time, place, registration, attention, and calculation, recall, language, copying | 490 elderly subjects aged >65 years,  170 were illiterate, 320 were educated, selected by cluster sampling method. | Cross sectional and analytical study to evaluate validity and reliability of r MMSE Turkish version and also reorganise the test and determine the cut off point for revised version. Validated against clinical diagnosis of dementia based on DSM IV. | - Cronbach’s alpha > 0.7  - In educated elderly correlations between intrarater and interrater test–retest was (0.966 (p<0.001); 0.855 (p<0.001), respectively).  -Kappa value for agreement between interraters (1.000) and intraraters (0.784) subjects.  - In uneducated elderly correlations between intrarater and interrater test–retest was (0.988 (p<0.001); 0.934 (p<0.001), respectively).  -Kappa value for agreement was (0.826) in interrater subjects,  and (0.656) in intraraters | In educated elderly Cut-off point  of 22/23 had the sensitivity(90.9) and specificity(97), positive predictive value(58.8), negative predictive value(99.6), positive likelihood ratio(30.3),  negative likelihood ratio(0.09).  In uneducated elderly cut-off point of 18/19 had the highest sensitivity (82.7), specificity (92.3) and  positive likelihood ratio (10.7). |
| Cognition  The short  cognitive test (SKT) Brazil version | Viola et al., 2011 | Flaks et al., 2009  Brazil  Portuguese | 9 subtests, scores fall between 0 and 27, with higher scores indicating  more severe cognitive impairment. Score tables are available for six age groups (17–44, 45–54, 55–64, 65–74, 75–84,  and 85 years and above) and three IQ levels (90, 90–110, and >110) | 184 patients of which 46 patients with AD, 82 with MCI, and 56 normal controls (NC). | Crosssectional study to check accuracy of SKT against Clinical diagnoses established at consensus sessions, by an expert multidisciplinary team, taking into account clinical,  neuropsychological, and neuroimaging data. | -AUC ranged from 0.7-1 for all subgroups like NC x MCI, NC x AD, MCI x AD | -sensitivity = 95.5%, 75% and specificity 50%, 53.7% for NC and MCI among subjects with >8 and <8 years of schooling respectively.  -Sensitivity = 100%, 100%, specificity 87.5%, 82% for NC and AD among subjects with >8 and <8 years of schooling respectively. |
| Cognition  Toronto alexithymia scale 20 Chinese version (TAS 20-Chinese) | Lin et al., 2015 | Zhu et al., 2007  China  Chinese | 20-item self-report instrument with each item rated on a 5-point Likert scale ranging from 1 (strongly disagree) to 5 (strongly agree); 5 items are inversely rated. scores range between 20 and 100, and higher scores mean a higher alexithymia. | 939 undergraduate students from a medical and non-medical university and 194 outpatients referred for assessment and treatment in a medical  psychological clinic | Crosssectional study to develop a Chinese translation of the  TAS-20 and to evaluate its reliability and factorial validity in both student and clinical samples and compare chinese data with Canadian sample (original site of TAS 20 development) | -Cronbach α coefficients for the full TAS-20-C were .79 for the student sample and .84 for the clinical sample.  -factor analyses yieled 3 factor model which showed best acceptable standards  -test retest coefficient >0.7 |  |
| Cognition  CERAD neuropsychological  Battery – Brazil version | Camargo et al., 2015 | Bertolucci et al., 2001  Brazil  Portuguese | AD battery consisting of tests -Verbal Fluency(animal category), abridged boston naming test(15 of the original 60 drawings), MMSE, word list memory, with repetition, recall and recognition, constructional praxis with copy and recall | Three groups,  -31 patients with mild AD  - 12 patients with moderate to severe AD  - 85 people aged 65 years and above without any memory problem | Cross sectional study to evaluate the applicability of the Portuguese Brazilian version of CERAD in Brazil and the differences between normal aged people and probable AD patients | Significant difference between the control group and patients with mild AD (p<0.0001) for all tests.  Difference between mild and moderate to severe AD were seen for MMSE, verbal fluency and boston naming (p<0.001) | Sensitivity and Specificity for verbal fluency (73.8 and 87.1), boston naming (61.9 and 69.4), MMSE (97.6 and 75.3), word list memory (85.7 and 87.1), constructional praxis (81 and 51.8), word list recall (74.2 and 82.4), word list recognition (76.2 and 87.1), praxis recall (87.1 and 67.1) respectively |
| Cognition  MMSE brazil | Camargo et al., 2015  Machado et al., 2009  Santos et al., 2015  Viola et al., 2011 | Lourenço et al., 2006  Brazil  Portuguese | 30 point questionnaire with questions to check orientation to time, place, registration, attention, and calculation, recall, language, copying | convenience  sample of 306 elderly patients attending an outpatient primary  care clinic at a university hospital in the city of  Rio de Janeiro, Southeastern Brazil | Cross sectional study to examine criteria validity  of the MMSE Portuguese version and validating it against diagnosis of dementia by geriatricians using structured interview based on DSMIV and ICD 10. |  | Sensitivity, specificity, positive and negative predictive values were 80.8%, 65.3%, 44.7% and 90.7% respectively for cut-off point of 23/24.  Area under ROC curve was 0.807 indicating good responsiveness |
| Cognition  CAMSE  Chinese adapted MMSE | Li et al., 2017  Lyu et al., 2018  Niu et al., 2010  Wang et al., 2018 | Xu et al., 2003  China  Chinese | 30 point questionnaire with questions to check orientation to time, place, registration, attention, and calculation, recall, language, copying | 370 elderly outpatients aged >60 years, from Xijing hospitals located in Xi’an, China, 93 of whom  were found to be demented and 277 non-demented | Crosssectional study to evaluate feasibility  of a Chinese adapted MMSE (CAMSE) for screening of  dementia among illiterate and less educated Chinese in outpatient clinical settings. | Test–retest reliability for total scores was 0.75 ( p<0.01). | Cut-off points of 22 for literates and 20 for illiterates yielded a sensitivity of 83.87% and a specificity of 84.48%.  Corresponding positive predictive value (PPV) was 0.65, and negative  predictive value (NPV) was 0.94 |
| Behavioural problems in Dementia  NPI – brazil | Novelli et al., 2018  Paddick et al., 2017  Viola et al., 2011 | Camozzato et al., 2008  Brazil  Portuguese | 12 behavioural symptoms, 3 sections for each symptom: frequency, severity, and  care givers distress. The total severity = (frequency x severity) score.  Total distress score calculated as the sum of the scores for each symptom.  The total NPI score is the sum of the subscales scores. | 36 AD patients and their  caregivers selected by consecutive referrals from the AD Center and Neurogeriatric Clinic from the Hospital de Clinicas de Porto Alegre, Brazil | Cross sectional study to evaluate the test-retest, inter-rater reliability and internal  consistency of the Brazilian Portuguese version of NPI and NPI-D and to determine the NPI profile in a sample of outpatients with AD in  southern Brazil. | -ICC= 0.98 (p=0.001) for the total severity score and 0.96 (p=0.001) for the total distress score.  - Cronbach’s *α* for both total severity and total distress reliability (across 12 domains) was 0.7. | - |
| Behavioural problems in Dementia  Neuropsychiatric inventory clinician rating scale (NPI-C) – Brazil version | De oliveira 2018 | Stella et al., 2013  Brazil  Portuguese | 12 domains. The clinician provides a  rating for each item in each domain based on interview with caregiver, direct observation and interaction with the patient, and additional information  from patient record | 312 participants (156 patient- informant  dyads) with the NPI-C totalling 624 observations in 5 Brazilian centers | Cross-sectional investigation of patients and respective caregivers to estimate inter-rater reliability and convergent validity of the Brazilian translation of NPI-C. | -inter-rater reliability(ICC) ranged between 0.777 and 0.947 for individual domains and 0.923 for total score | -Strong correlation between most NPI-C domains and the  selected validation scales.  -The highest correlation was observed between NPI-C/Apathy  domain and Apathy Inventory (0.942) |
| Behavioural problems in Dementia  Chinese Neuropsychiatric inventory(CNPI) | Li et al., 2017  Lyu et al., 2018  Niu et al., 2010 | Wang et al., 2012  China  Chinese | 12 factors of behavioural problems-delusions, hallucinations,  agitation/aggression, depression, anxiety, euphoria,  apathy, disinhibition, irritability, abnormal motor  behavior, nighttime behavior, appetite and eating disorders. Each  factor is evaluated in terms of severity and  frequency. Total score range is 0–120 points. Higher scores represent  more severe psychiatric symptoms. | 219 caregivers of patients with AD. All AD subjects were recruited from the tertiary hospital of Shanghai Mental Health Center, Shanghai. | Crosssectional study to evaluate the reliability and validity of the  CNPI. | For CNPI symptom sub-questionnaire  -The Cronbach’s α= 0.69.  - Test–retest correlation coefficient =0.66 -0.95 (p<0.001)  -correlation coefficient for the total symptoms questionnaire score was 0.96 (p<0.001)  For caregiver distress  sub-questionnaire  -The Cronbach’s α= 0.72.  - Test–retest correlation coefficient 0.66 -0.98 (p<0.001)  - correlation coefficient for the total caregiver distress scores was 0.94 | Principal axis factoring analysis of  the symptom subscale and caregiver distress subscale yielded a five-factor solution each, which contributed to 67.0% and 70.2% of the cumulative variance respectively.  -results indicate that the CNPI provides  good internal construct validity |
| Anxiety and depression  Hospital anxiety and depression scale (HADS)  Self rating Chinese version | Lin et al., 2015 | Leung et al., 1999  China  Chinese Cantonese | 14 item scale, with 2 sub scales for anxiety and depression with 7 items each.  Each item scored from 0-3. Score range from 0-21 for either depression or anxiety | A total of 93 subjects were recruited over a period  of 6 months from adult in-patient consultation referrals from different  specialties to the Department of Psychiatry at Prince of Wales Hospital, Hong Kong | Cross sectional study to validate the Chinese-Cantonese  version of the HADS and to determine the optimal cut-off  points and respective specificity and sensitivity, and to compare with the Hamilton Rating Scale of  Depression (HRSD), and Hamilton  Rating Scale of Anxiety (HRSA) | -Cronbach’s alpha, was 0.86 for the full scale, 0.82 for the depression subscale and 0.77 for the anxiety subscale.  -The mean inter-item correlation was 0.30 for the full scale, 0.41 for the depression subscale and 0.33 for the anxiety subscale.  -The item-scale correlation was 0.60  for the full scale, 0.70 for the depression subscale  and 0.66 for the anxiety subscale | -Pearson’s coefficient=  0.67 (with HRSD) and 0.63 (with HRSA), P<O.OOl).  -Both subscales showed a similar correlation with the two Hamilton scales (range 0.52-0.59; P<O.OOl.  -optimal cut-off point of 15/16 for the full scale yielded a sensitivity of 0.79 (95% CI=O.66-0.90) and a specificity of 0.80 (95% CI=O.69-0.91). |
| Depression  GDS-30 Brazil | Machado et al., 2009  Viola et al., 2011 | Castelo et al., 2010  Brazil  Portuguese | 30 item scale, with yes or no options,  0-9 : normal  10-19: mild depression  20-30: severe depression | 220 elderly patients aged 60 years and above recruited from four primary care clinics in north eastern Brazil. | Cross-sectional study to determine the validity of the Brazilian version of the GDS and calculate the optimum cut off points for identifying depression among elderly primary care subjects. Diagnosis compared with Portuguese version of DSM IV -SCID1 evaluation by a psychiatrist. | -Cronbach’s *α=* 0.88; (95% CI: 0.86–0.90) demonstrated good internal consistency reliability. | -Cut-off score of 10/11 yielded sensitivity- 92.1% (95% CI: 77.5–97.9); specificity- 79.7% (95% CI: 72.9–85.1); PPV -48.6% (95% CI: 36.8–60.3); and NPV 98.0% (95% CI: 93.7–99.5). |
| Depression  GDS – 15 Brazil | Santos et al., 2015 | Paradella et al., 2005  Brazil  Portuguese | 15 item scale derived from GDS-30 | 302 patients aged 65 years and above in a public outpatient clinic in the city of Rio de Janeiro, Brazil | Cross-sectional validation study of GDS 15 against medical evaluation by geriatricians using by the Portuguese version of the Structured Clinical Interview for DSM-IV (SCID-I) | -cutoff value of 5/6, the area under the ROC curve was 0.85 (95% CI: 0.79–0.91). | -Cut off value of 5/6 had sensitivity of 81% and specificity of 71% |
| QOL  RAND 36/ Short form health survey/ SF36 Chinese version | Lin et al., 2015 | Li et al., 2003  China  Chinese | 36 itemquestionnaire, consists of 8 scaled scores, which are the weighted sums of the questions in their section. Each scale is directly transformed into a 0-100 scale on the assumption that each question carries equal weight. lower the score the more disability | 1000 households in 18 communities of Hangzhou. 1688 respondents recruited by multi-stage mixed sampling. 57 subjects were randomly sampled for test-retest  study after two weeks | Crosssectional study to develop a self administered Chinese (mainland) version of the Short-Form Health Survey (SF-36) for use in health related quality of life measurements in China | -Two weeks test-retest reliability coefficients ranged from 0.66 to  0.94  -Cronbach’s alpha ranged from 0.72 to 0.88 except 0.39 for the social functioning  scale and 0.66 for the vitality scale | -Factor analysis identified two principal components explaining 56.3% of the total variance.  -scaling success rates were 91.4% (32 of 35) for convergent validity, and 92.5% (259 of 280) for discriminant  validity |
| Quality of life  WHOQOL bref | Mkenda et al., 2018  Paddick et al., 2017 | WHOQOL group 1998  Tanzania  English | 26 questions rated on a 5 point scale, 24 questions across 4 domains and 2 questions that address overall quality of life and general health.  4 domains: physical health, psychological, social relationships and environment. Each domain score range from 4-20. The raw score of each domain is calculated by multiplying mean of each domain by 4 for compatibility  with the scores used in WHOQOL-100. | 300 people approx. from each study site.  WHOQOL pilot study – 15centres (n=4802), field testing at 13 centres (n=3882), new phase- 5 centres (n=2369) | A multiphase, multisite Cross section study to develop WHOQOL bref from WHOQOL-100 which was developed from its original 236 item questionnaire. | -Cronbach alpha values for each of the four domain scores ranged from 0.66 - 0.84  -test-retest reliabilities for domains were  0.66 for physical health, 0.72 for psychological,  0.76 for social relationships and 0.87 for environment. | -high correlations ranging between 0.89-0.95 for domain scores based on the WHOQOL-100 and WHOQOL-BREF domain scores.  - WHOQOL-BREF was shown to be comparable  to the WHOQOL-100 in discriminating between ill and well subjects, with similar values and significant correlation  apparent in all domains. |
| Quality of life  QOL- AD | Machado et al., 2009  Santos et al., 2015  Novelli et al., 2018  Viola et al., 2011 | Novelli et al., 2010  Brazil  Portuguese | 13 items quantified on a scale ranging from 1(poor) to 4(excellent), total score ranges from 13 to 52. 3 versions. Patients' and caregivers' reports on patients' quality of life, caregivers' reports on their own QOL (PQOL , C-PQOL and CQOL respectively. | 120 individuals, 30 patients with mild AD, 30 patients with moderate AD and their caregivers/  Family members (n=60) participated in this study. | Cross sectional study to assess the reliability and construct validity of the Brazilian version of the QOL scale in AD. Data was compared with WHOQOL-Bref scores (gold standard) | Cronbach's alpha for PQOL - 0.80,  C-PQOL- 0.83, and CQOL -0.86 shows high reliability. | - The correlation coefficients were higher than  0.70 for total score on all the 3 versions of QOL-AD scale with the total score of the WHOQOL-brief. |
| Quality of life  WHOQOL bref Hindi | Kumar et al., 2014 | Saxena et al., 1998  India  Hindi | 26 questions rated on a 5 point scale, 24 questions across 4 domains and 2 questions that address overall QOL and general health.  4 domains: physical health, psychological, social relationships and environment. Each domain score range from 4-20. The raw score of each domain is calculated by multiplying mean of each domain by 4 for compatibility with the scores used in WHOQOL-100. | 304 adult subjects in Delhi. (253 persons with disease or impairment; 51 well  persons) selected by purposive sampling from patients and relatives visiting AIIMS, Delhi respectively | The pilot field trial using the 236-ltem questionnaire to develop a QOL  instrument (WHOQOL-Hindi) in collaboration with the WHOQOL  Group, Division of Mental Health and Prevention of Substance  Abuse, World Health Organization, Geneva. | -Cronbach alpha for each domain ranged between 0.59-0.91  -All facets correlated significantly with their respective domains.  -All the facets and domains also correlated significantly with the  overall QOL score.  -all items distinguished significantly between the 'diseased' and the 'healthy'. | - Domain scores produced by the WHOQOL-Bref have been shown  to correlate at around 0.9 with the WHOQOL-100 domain scores. |
